# Content Validity of An Aging-related Preparation Education Program For Older Chinese Migrants In Japan

**DOI:** 10.1093/geroni/igaf122.2596

**Published:** 2025-12-31

**Authors:** Li Yao, Harue Masaki

**Affiliations:** Chiba University, Chiba, Chiba, Japan; Chiba University, Chiba, Chiba, Japan

## Abstract

**Introduction and Objectives:**

This study aimed to verify the content validity of the developed aging-related preparation education program through experts in gerontological nursing. This program was provided to older Chinese migrants in Japan, focusing on long-term care, and included six modules with lectures and discussions conducted via real-time videoconferences.

**Methods:**

Nursing researchers specializing in gerontology and care providers experienced in caring for older Chinese migrants were recruited. Participants were asked to review the developed education program materials, including a booklet and six lecture videos for each module. They then partook in semi-structured interviews based on the questionnaire responses. The questionnaire focused on assessing content clarity, structure appropriateness, and consistency of the instructional content and methods using a 4-point Likert scale. Descriptive statistics were used to analyze questionnaire responses, while content analysis was used to analyze the interview data.

**Results:**

Three Chinese gerontological nursing researchers and two Chinese practicing care providers in Japan were enrolled in this study. Regarding the appropriateness of instructional content and methods, all participants evaluated modules No. 1-4 as ‘appropriate’ or ‘somewhat appropriate’, while 20% evaluated modules No. 5-6 as ‘somewhat inappropriate’. Seven categories related to the evaluation reasons and revision suggestions were identified from the interview data.

**Conclusion:**

This education program was valid for older Chinese migrants in Japan, with easy-to-understand booklets and module slides, and was appropriate in terms of educational goals, structured instructional content, and volume. Moreover, case introductions through videos or photographs were requested to offer comprehensive content.

